# Hemispheric Asymmetry of Intracortical Myelin Orientation in the Mouse Auditory Cortex

**DOI:** 10.1111/ejn.16675

**Published:** 2025-01-20

**Authors:** Philip Ruthig, Gesine Fiona Müller, Marion Fink, Nico Scherf, Markus Morawski, Marc Schönwiesner

**Affiliations:** ^1^ Faculty of Life Sciences Leipzig University Leipzig Germany; ^2^ Paul Flechsig Institute–Centre of Neuropathology and Brain Research, Medical Faculty University of Leipzig Leipzig Germany; ^3^ IMPRS Neurocom Max Planck Institute for Human Cognitive and Brain Science Leipzig Germany; ^4^ Faculty of Computer Science TU Dresden University of Technology Dresden Germany; ^5^ Methods and Development Group Neural Data Science and Statistical Computing Max Planck Institute for Human Cognitive and Brain Sciences Leipzig Germany; ^6^ Department of Psychology Université de Montréal Montréal Canada

**Keywords:** auditory cortex, cortical columns, lateralization, light sheet microscopy, myelination, vocalization

## Abstract

Communication sound processing in mouse AC is lateralized. Both left and right AC are highly specialised and differ in auditory stimulus representation, functional connectivity and field topography. Previous studies have highlighted intracortical functional circuits that explain hemispheric stimulus preference. However, the underlying microstructure remains poorly understood. In this study, we examine structural lateralization of AC on the basis of immunohistochemically stained and tissue‐cleared adult mouse brains (*n* = 11). We found hemispheric asymmetries of intracortical myelination, most prominently in layer 2/3, which featured more intercolumnar connections in the right AC. Furthermore, we found a larger structural asymmetry in the right AC. We also investigated sex differences. In male mice, myelination direction in the right AC is tilted to the anterior side. This pattern is inverted in female mice. However, the spatial distribution of neuronal cell bodies in the left and right AC along the laminar axis of the cortex was remarkably symmetric in all samples. These results suggest that basic developmentally defined structures such as cortical columns remain untouched by lateral specialisation, but more plastic myelinated axons show diverse hemispheric asymmetries. These asymmetries may contribute to specialisation on lateralized tasks such as vocal communication processing or specialisation on spectral or temporal complexity of stimuli.

## Graphical abstract

We find that the right and left auditory cortex in mice are both organised in cortical columns, with asymmetric myelination in the different layers and differences between the sexes. In addition, the right auditory cortex shows greater intercolumnar connectivity than the left.

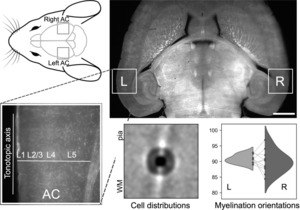

**Abbreviations**
AC: auditory cortex
VC: visual cortex

## 1 Introduction

The mammalian brain is inherently asymmetric (Mundorf and Ocklenburg 2023; Rivera‐Olvera et al. 2024). Both mice and humans display various asymmetries, for example, in the motor system (Manns et al. 2021) and in acoustic communication (Friederici and Gierhan 2013; Ocklenburg, Ströckens, and Güntürkün 2013). A laterally asymmetric organisation of brain regions allows the brain to represent information in a more efficient way (Vallortigara 2006) and at the same time allows for a certain degree of redundancy, for example, in the case of stroke (Saur et al. 2006). Therefore, hemispheric asymmetry is very evolutionarily successful in mammals and was even documented in birds (Casey and Martino 2000; Güntürkün et al. 2000) and some invertebrates (Halpern et al. 2005). In this paper, we focus on the early auditory system, where left and right auditory systems preferentially represent stimuli depending on their spectral and harmonic properties (Poeppel 2003; Schönwiesner, Rübsamen, and Yves Von Cramon 2005; Zatorre, Belin, and Penhune 2002). For a more comprehensive review of lateralized auditory cortex (AC) function, see Ruthig and Schönwiesner (2022).

Mice ( *Mus musculus* ) are social mammals and use temporally and harmonically complex vocalisations to communicate with their conspecifics. The acoustic units of these vocalisations are short syllable‐like units of varying acoustic complexity, from relatively simple up or down sweeps to more complex phonetic units with multiple formants (Arriaga, Zhou, and Jarvis 2012). These units are concatenated to phonetic units, reminiscent of birdsong (Holy and Guo 2005). To produce and interpret these calls, mice have vocal abilities and neuroanatomical features long thought to be exclusive to primates and songbirds (Arriaga, Zhou, and Jarvis 2012). Mouse calls can convey information such as sex, caller identity and emotional state (Seyfarth and Cheney 2003) and are used to form long‐lasting relationships (Laham, Diethorn, and Gould 2021), negotiate territorial encounters (Portfors and Perkel 2014) and relay information about the affective state of infants. The specific structure of vocalisations depends on social context, such as intersexual presence and the estrous cycle (Chabout et al. 2015; Gaub, Fisher, and Ehret 2016; Hanson and Hurley 2012).

These vocalisations are analysed preferentially in the left AC of mice. Behaviourally, this lateralization is detectable as a right ear advantage for social stimuli (Ehret 1987; Ehret and Geissler 2006). The left AC is larger than the right (Stiebler et al. 1997) and more active during exposure to vocalisations (Levy et al. 2019). The left AC is also relatively more active in response to pup calls (Geissler, Schmidt, and Ehret 2016) and other oxytocin‐dependent behaviours (Marlin et al. 2015; Mitre et al. 2016; Tasaka et al. 2020).

The representation of vocal stimuli in the mouse AC is complex. Left and right AC are roughly tonotopically organised (Romero et al. 2020; Stiebler et al. 1997), although neighbouring neurons often have very different characteristic frequencies (Issa et al. 2017; Maor, Shalev, and Mizrahi 2016). AC neurons form dynamic networks that change over the course of days (Betzel et al. 2019), and most stimuli are only represented in < 5% of neurons (Hromádka, DeWeese, and Zador 2008). These representations likely form spontaneously on stimulus presentation (Shiramatsu et al. 2016). One specific mechanism by which these representations might be formed was proposed by Levy et al. (2019), who suggest that specific lateralized feature detectors are responsible for bottom‐up feature selectivity in the AC (reviewed in Neophytou and Oviedo 2020). Although the functional and structural lateralization of AC is evident, the underlying microstructure remains unclear. Does AC microstructure reflect the observed functional lateralization? Given that the context of acoustic communication is very dependent on the sex of the listener, are sex‐dependent differences present in both structure and function? Despite these open questions, detailed studies of both ACs remain rare. Typically, only a single hemisphere is investigated (reviewed in Ruthig and Schönwiesner 2022). To generate biologically realistic functional models of AC, an assessment of the underlying cellular structure and structural specialisations in both ACs is critical.

In this study, we address these open questions by investigating the left and right AC of healthy adult mice with optical clearing coupled with light sheet fluorescence microscopy (Huisken et al. 2004). We describe lateralized myelinated axonal structures and non‐lateralized spatial features in the distribution of neuronal cell bodies in the AC.

## 2 Materials and Methods

### 2.1 Animals

We used 11 (6 female, 5 male) adult (p35‐p37) C57BL/6J‐Tg (Thy1‐GCaMP6f)GP5.11Dkim/J mice, which were kept in the animal facility of the faculty of Life Sciences of University Leipzig. All experiments were conducted according to the regulations of the Landesdirektion Sachsen (§ 4 Abs. 3 TierSchG, Versuchsvorhaben T13/19).

### 2.2 Perfusion

The mice were injected with Heparin (0.1 mL/50 g body weight) intraperitoneally and sit for 5 min. The animals were then deeply anaesthetised with CO_2_ and transcardially perfused with 3.6 mL/min of 0.9% NaCl for 5 min and afterwards with 4% paraformaldehyde in PBS for 20 min. The brains were extracted directly after perfusion and kept in 4% paraformaldehyde in PBS at 4°C for 9 days for passive fixation.

### 2.3 Staining

Staining and clearing was performed analogous to iDISCO+ procedure described by Renier and colleagues (Renier et al. 2016), except with longer immunostaining incubation times and an additional refreshment of primary antibody. During all incubation times, the samples were in glass vials on a shaker shaking at 70 rpm. The samples were dehydrated in MeOH/PBS (50%–80%–100% for 1.5 h each, shaking at room temperature), bleached with H_2_O_2_ (6% H_2_O_2_ + MeOH overnight at 4°C in the dark), then rehydrated in MeOH/PBS (100%–80%–50% for 1.5 h each, shaking at room temperature). Blocking was done with PBSGT (PBS + 0.2% Gelatine + 0.5% Triton‐X 100 + 0.1% NaN_3_) at room temperature for 12 days. Incubation with both primary antibodies anti‐human HuC/HuD (Thermo Fisher Scientific HuC/HuD (Catalogue # A‐21271), reconstituted in 0.5 mL PBS + 1% BSA + 0.1% sodium azide, 1:400) and anti‐rat? MBP (Myelin Basic Protein by Abcam (Catalogue # Ab7349), 1:400) in PBSGT was performed for 22 days at 37°C. We refresh the staining solution incubation after 11 days to refresh potentially degraded components. After washing with PBSGT (refreshed 6× daily at 1 h intervals, shaking) for 3 days at room temperature, the samples were incubated with secondary antibodies Cy3 polyclonal donkey anti‐mouse (Jackson ImmunoResearch, 1:1000), Dylight 755 polyclonal donkey anti‐rat (Invitrogen, 1:1000) and nuclear staining TO‐PRO‐3 (Invitrogen, 1:1000) in PBSGT for 13 days at 37°C. After that, samples were once again washed with PBSGT (refreshed 6× daily at 1 h intervals, shaking) for 3 days at room temperature. For details of (immuno‐)staining compounds, see Table S1.

### 2.4 Clearing

We conducted iDISCO+ clearing analogous Renier and colleagues (Renier et al. 2016). During all incubation times, samples were on a shaker shaking at 40 rpm. Samples were dehydrated in MeOH/PBS (20%–40%–60%–80%–100%–100%) for 1 h each at room temperature, shaking. After that, samples were incubated in 66% dichloromethane and 33% MeOH solution for 3 h. Then, they were incubated in 100% dichloromethane for 2 h, refreshing dichloromethane after 1 h, shaking. To initiate the clearing procedure, the samples were incubated in BABB until transparent (not shaking from this point on). After clearing, the samples are kept in BABB at room temperature until image acquisition. While immunostained and cleared samples can typically be kept for several months at least, the myelin basic protein staining showed visible degradation over the following weeks. Therefore, we imaged 1–3 days after clearing. For details regarding the chemicals used, see Table S2.

### 2.5 Acquisition

The data were acquired with a LaVision Biotec Ultramicroscope II with a Neo 5.5 sCMOS camera, with BABB as imaging medium and a MI Plan 12× objective (NA = 0.53, voxel size 0.54 × 0.54 × 4 μm XYZ). This is a sufficient resolution to reliably detect cells in the data but does not allow segmentation of single fibres. Image data were acquired in a 2 × 3 stack mosaic acquisition, including primary auditory and primary visual cortex and surrounding areas. Light sheet NA was set to 0.165 (minimum sheet thickness 3.4 μm), with a sheet width of 40% (~1.2 cm) and an exposure time of 97 ms. Laser power for the respective lasers (as defined by the laser source) was set to 7.5 (488 nm), 22.5 (555 nm), 3.25 (640 nm) and 75 mW (785 nm). To minimise a potential bias from the optical system, all samples but one were scanned with light sheets from the right side.

### 2.6 Preprocessing

Before further analyses, the data were re‐saved from raw 2D TIFF stacks to 3D Multi‐Channel TIFF stacks of the Auditory and Visual Cortex, respectively. The script is included in the data repository on BioImage Archive and the code repository (see Data Availability Statement).

### 2.7 Annotation of Neurons

We convolved our raw data with a custom three‐dimensional Gabor spherical shell kernel (See Figure S3) to enhance local contrast. The kernel is based on a previously described circle detection method (Rhodes and Bai 2011) with a third dimension to create a lens‐shaped edge detection kernel (“gabor spherical shell”). This kernel consists of a gaussian envelope with the standard deviation σ, which is offset by a certain radius r around the origin of the kernel $r_{o}=\left(x_{0},y_{0},z_{0}\right)$. The Gaussian envelope is modulated by a complex plane wave originating from $\left(0,0,0\right)$ expanding radially outwards with its respective frequency $f_{0}$ and phase shift φ. The Gabor spherical shell kernel is therefore defined as follows:

$$K_{r}K_{i}\left(x,y,z\right)=\frac{1}{2\pi \sigma r_{o}}e^{-\pi \left[\frac{{\left(r-r_{0}\right)}^{2}}{\sigma^{2}}\right]}e^{i\left[2\pi f_{0}\left(r-r_{0}\right)+\varphi \right]}$$

where

$$r=\sqrt{{\left(x-x_{0}\right)}^{2}+{\left(y-y_{0}\right)}^{2}+{\left(z-z_{0}\right)}^{2}}$$

$K_{r}$ and $K_{i}$ are the real and imaginary parts of the kernel. We convolved the real part of the kernel with the image data using SciPy's fast Fourier transform convolution (Virtanen et al. 2020). The convolution highlights spherical features in the image, which were measured by detecting and marking local maxima for further analysis. The Z dimension of the Gabor spherical shell kernel was compressed to account for the Z anisotropy of the microscopy data.

### 2.8 Cell Distribution Analysis

All detected cell centres *n* were labelled with an index i, where i=1,2,3…n. For a given neuron c, a three‐dimensional grid is placed over the surrounding tissue with its origin $\left(0,0,0\right)$ at the active neuron $c_{i}$ and the edge length d. The number of surrounding marked cell centres is measured, resulting in a local cell density field $p_{i}\left(x,y,z\right)=c_{i}\left(x,y,z\right)/d^{3}$. This process is repeated for a subset of all cells ($c_{i}$ with $i\;\epsilon \;\left\{5,10,\ldots n\right\}$). The average neuronal density field is then computed across AC samples (hemispheres and animals) as ${\Sigma^{n}}_{i=1}\left(x,y,z\right)/n$. For the analysis presented here, an edge length d=54μm is used. To prevent edge artefacts, only cell centres at least 54 μm from the edge of the images were selected for analysis.

### 2.9 Quantifying Cortical Fibre Orientations

Local orientation features are valuable cues for quantitative analysis of image data, especially in biological and medical applications. Here, we use the orientation of intracortical myelinated neurons to learn about structural differences between the left and right AC in mice. Orientations are determined by computing the local structure tensor at each pixel position and averaging those in a local window to define dominant directions (Morawski et al. 2018). A tailored analysis pipeline is established (Figure S6) and results in local dominant orientations are fitted with circular regression models. Due to low staining contrast because of poor antibody penetration in cortical layer 6, we exclude this layer from the analysis.

The tailored orientation analysis uses the MBP and autofluorescence channel to compute in‐plane local dominant directions using a 2D sliding window approach. Following a median and Sato filter, the MBP channel is normalised, and the structured tensor is computed for each pixel in a sliding window of size 24 × 24 pixels. The structure tensor J can be interpreted as a covariance matrix of the localised image gradient, and its eigenvalues λmax, λmin depict the strength of homogeneity and type of orientation in that local window (Van Der Walt et al. 2014). Additionally to the orientation ϕ, the energy E representing homogeneity when E ≈ 0 (λmax = λmin≈0) and the coherence C being a measure of confidence if E ≫ 0 can be calculated. C equals 1 if one distinct dominant direction can be found and equals 0 if the structures are essentially isotropic (Püspöki et al. 2016).

$$\text{Orientation }\phi =\frac{1}{2}\arctan\left(\frac{2J_{12}}{J_{22}-J_{11}}\right)$$

$$\text{Energy}\;E=\text{trace}\left(J\right)$$

$$\text{Coherence }C=\frac{\lambda_{\text{max}}-\lambda_{\text{min}}}{\lambda_{\text{max}}+\lambda_{\text{min}}}\in \left[0,1\right]$$

The resulting orientation value is stored if the coherence values multiplied with the energy is not below a predefined threshold. This procedure is repeated for all in‐plane windows and for each plane in every dataset.

The AF channel is processed to define the location of each dominant direction with respect to the cortical surface and thus its belonging to a particular cortical layer. The cortex surface is approximated by a quadratic function, and a gradient filter is applied to a distance transform of the resulting binary image. Then, the dominant directions are corrected according to the cortex surface curvature using the gradient filter over the distance transform. The cortical layer identity of every orientation value is stored. The analysis was not performed in 3D because of the high Z‐anisotropy of the data, which is typical of most commercial light sheet microscopes. We chose to orient the Z axis of our data along the dorsal‐ventral axis because none of the hypotheses we tested depended on structural variation along this axis. This means that we can focus on structural orientations of fibres in the XY plane, taking into account the cortical layers and the standard position of the tonotopic axis.

### 2.10 Bayesian Modelling of Intracortical Fibres

The orientation values are modelled quantitatively by an embedded regression approach using projected normal circular general linear models. This allows the assessment of potential angular differences between the cortical orientations of the two AC sides, taking into account the circular nature of the data. In the indirect modelling approach, as opposed to direct modelling, the circular outcome variable is defined by a bivariate linear space that must be translated back to the circular input space. To do this, we use the projected normal distribution, which treats the true input distribution as latent, and try to model it using Bayesian methods and an MCMC sampler. The R package bpnreg is used (Cremers, Mulder, and Klugkist 2018; Nuñez‐Antonio and Gutiérrez‐Peña 2014). We fit two different models: the first model included only the hemispheric side (L, R) as an explanatory variable and the second model included the side and the cortical layer identity.

For an effect to be considered distinct from another, the highest posterior density intervals (HPD) must not overlap. The HDP describes the interval in which 95% of the parameter values lie, or in other words, the probability that the effect lies between the lower and upper bounds is 0.95.

The use of R and the bpnreg package implementation was limited by the amount of data that could be included in a modelling instance. As we have approximately 6 million data points including all samples, the size of the matrix required for the modelling approach exceeded the memory limits. We solved this by bootstrapping the input data and running multiple sub‐models on individual mice and sex groups.

For the bootstrapping approach, subsamples are randomly drawn from the full datasets, but equal sizes for the model parameters are respected. Then, the analysis is performed on the subsampled data. Subsample sizes were chosen such that the distribution of the full dataset is recovered. This procedure is repeated 25 times, giving some confidence in the projected means and HPD intervals.

## 3 Results

### 3.1 Myelination Within Auditory Cortex Is Lateralized

**FIGURE 1:**
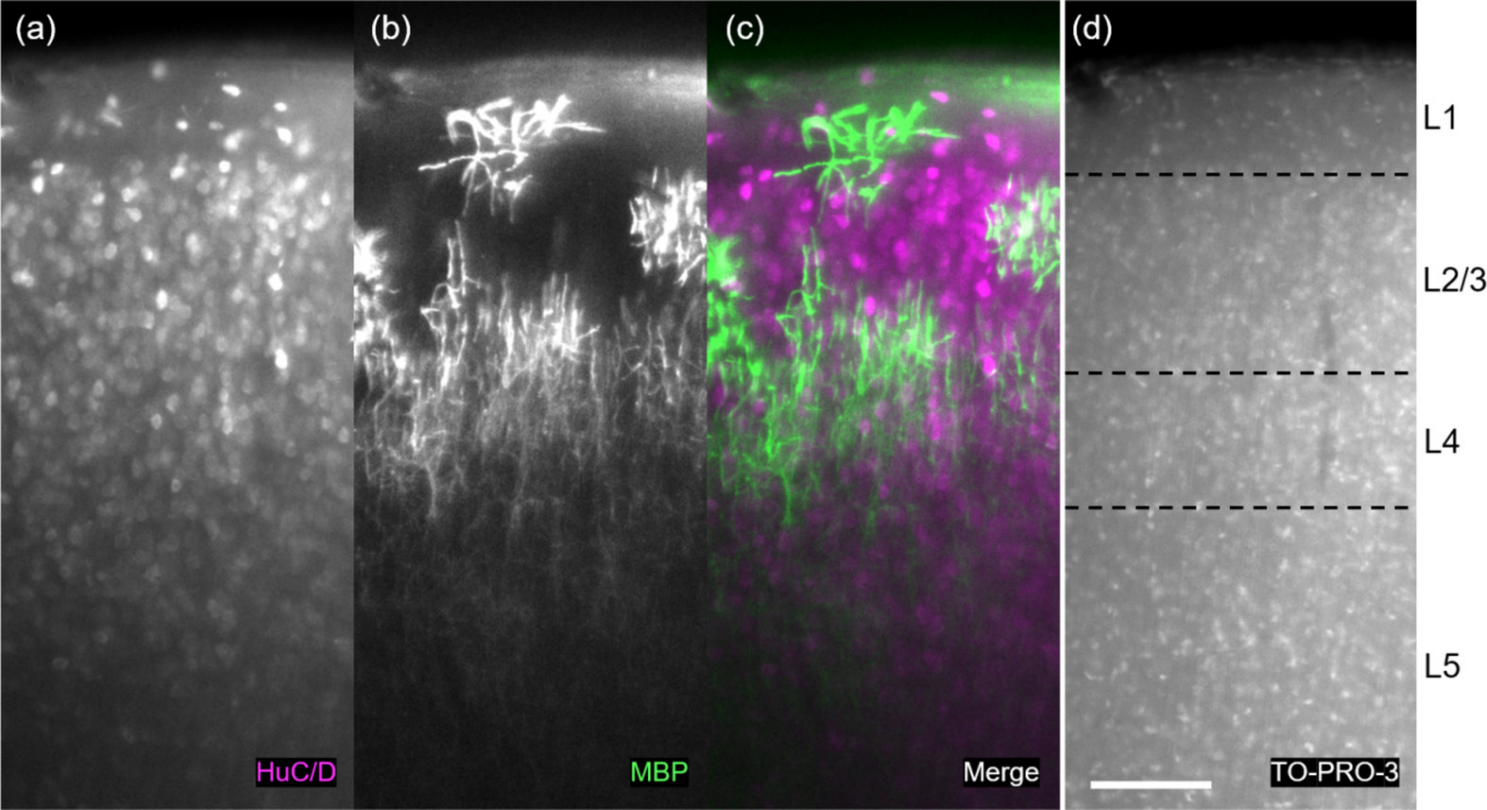
Stained 2D raw sections of tissue‐cleared AC imaged with light sheet microscopy. (a) HuC/HuD staining showing neuronal cell bodies, (b) myelin basic protein (MBP) staining showing myelinated axons and (c) composite of (a) and (b). (d) TO‐PRO‐3 staining used to define cortical layers (see Figure S2). Scale bar 200 μm.

We present evidence for layer‐specific lateralization in the AC of adult mice. We image cell body and fibre positions in immunohistochemically stained and optically cleared left and right AC (Figure 1). Light sheet microscopy allows optical sectioning by selectively illuminating a thin plane within the sample and capturing full‐frame images from an orthogonal detection axis (Huisken et al. 2004). We analyse these data by quantifying local orientation of myelinated axons in AC and use visual cortex (VC) as a control region. We performed circular regression of dominant directions based on myelinated axonal structures either on the whole datasets with all mouse samples together or on each sample individually. This allows some assessment of sample variation. Cortex lateralization can be recovered structurally with a small but measurable effect between the two hemispheres and between cortical layers. Furthermore, we found an effect of sex on the lateralization of myelin structure in AC.

**FIGURE 2:**
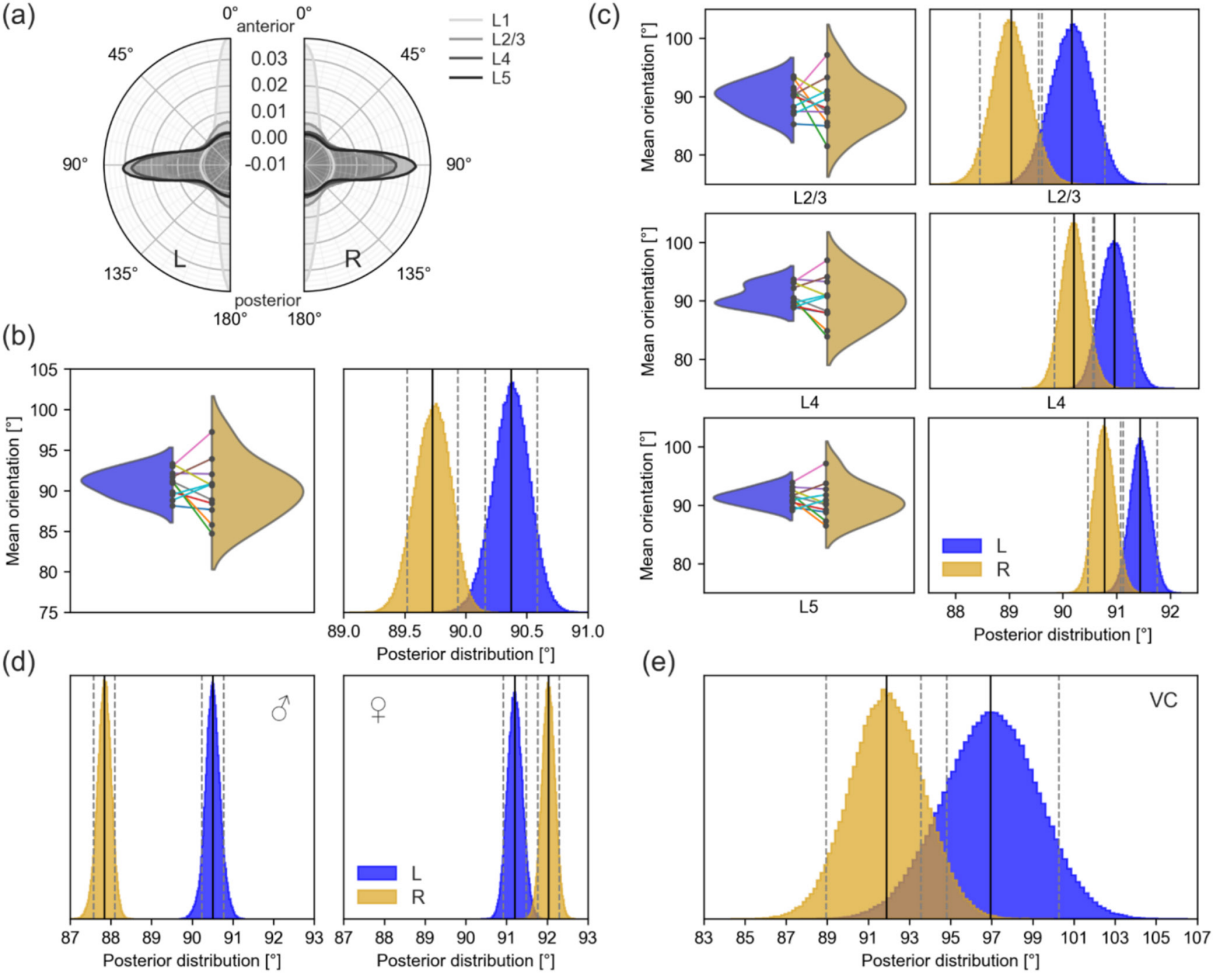
Hemispheric differences in fibre orientation in mouse AC. (a) The distribution of all dominant directions for all samples and included cortical layers (L1–L5) for the left and right AC, respectively. (b) Results of the circular regression model with only the side (left or right) as an explanatory variable are shown. In the violin plots, the means of the posterior distributions assessed for each sample individually are depicted, and the histograms show the posterior distributions of the circular regression model for all samples together. In the violin plots, individual sample means for the left and right AC are connected with lines. (c) Results of the circular regression model with the side (left or right) and the cortical layer identity as an explanatory variable are shown. Violin plots and posterior distributions of the circular regression model are given for each layer individually. For the violin plots, the regression was performed on each sample individually. Sample means are connected by lines. For the posterior distributions, the regression was done on all samples together. (d) Posterior distributions of the circular regression models with only the side as the explanatory variable, split by sex. (e) Posterior distributions of the circular regression model with only the side as the explanatory variable, using left and right visual cortex (*n* = 5) as control regions. All angular values are given in degrees, ranging from 0° to 180°. For all posterior distributions, the vertical lines represent the mean (black) and indicate the lower and upper bounds of the 95% highest density posterior (HDP, grey).

We tested two different model complexities using an embedded regression approach with projected normal circular general linear models. The first model included only the hemispheric side (L, R) as an explanatory variable. The second model included the side and the cortical layer identity. We used the deviance information criterion (DIC) to compare the model fit for the two different model complexities to judge the effect of including these additional factors in the regression model: The lower the DIC, the better is the model fit. In comparison to the first model, the DIC drops dramatically when we include the cortical layer identity (from 343,396 ± 949 to 162,503 ± 689). Since the exact localisation of the tonotopic axis can vary dramatically (as shown in, e.g., Francis et al. 2018) and has not been functionally determined beforehand, we did not include the tonotopic axis as an additional parameter. The model using only the hemispheric side as an explanatory variable shows that the difference between the left and right AC is approximately 0.7° (Left: 90.9° ± 0.1°, Right: 90.2° ± 0.1° [mean ± sd]). The differences in the left and right AC when additionally including the cortical layers results in values for each cortical layer separately: In L2/3, the hemispheric difference in the AC is 1.1° (L: 90.1 ± 0.2, R: 89.0 ± 0.2), in L4 0.7° (L: 90.9 ± 0.2, R: 90.2 ± 0.1) and in L5 0.6° (L: 91.4 ± 0.1, R: 90.8 ± 0.1). The largest difference is in L2/3, indicating that differences between the left and right AC can most likely be recovered in L2/3. Furthermore, L2/3 is different from L4 and L5 due to non‐overlapping highest posterior density intervals (HPD), but L4 and L5 are not. The mean orientation in L2/3 is significantly different from L4 and L5 (95% HPD not overlapping, Figure 2c) in the right and left AC. Additionally, the distribution of dominant directions is wider in L2/3 (left: 1.3°, right: 1.2°) than in L4 (0.7°) and L5 (left: 0.7°, right: 0.6°) based on the 95% HDP (Figure 2c). In L1, most myelinated axons are oriented in parallel to the cortical surface (Figure 2a). When comparing the asymmetries we describe in AC to VC (our control region in this case), we find no such difference in the VC. The HPDs are much wider and overlapping.

We observed large inter‐individual differences in the dominant fibre direction (coloured lines in Figure 2b,c). Since the interpretation of vocal communication can be highly sex‐dependant (e.g. mating calls), we tested if the sex of individuals influences lateralization of the mean orientation of axons in AC. We find differences in AC lateralization between males and females (Figure 2d). This can be recovered in both model complexities. Employing the regression model using only the side as explanatory variable and splitting the analysis between male and female results in orientation differences for the male mice of 2.7° (L: 90.5 ± 0.1, R: 87.8 ± 0.1) and for the female mice of −0.8° (L: 91.2 ± 0.1, R: 92.0 ± 0.1). The difference between the male and female groups is predominantly in the right AC, with a shift of 4.2° from male to female, whereas the shift on the left AC is 0.7° from male to female. Furthermore, the posterior distributions are slightly broader for the female group (Figure 2d). We also show distributions of dominant directions (analogous to Figure 2a) split into anterior, middle and posterior sections of the AC and split by sex (Figure S5).

### 3.2 Columnar Microstructure Is Present in AC, but Is Not Hemispherically Asymmetric

**FIGURE 3:**
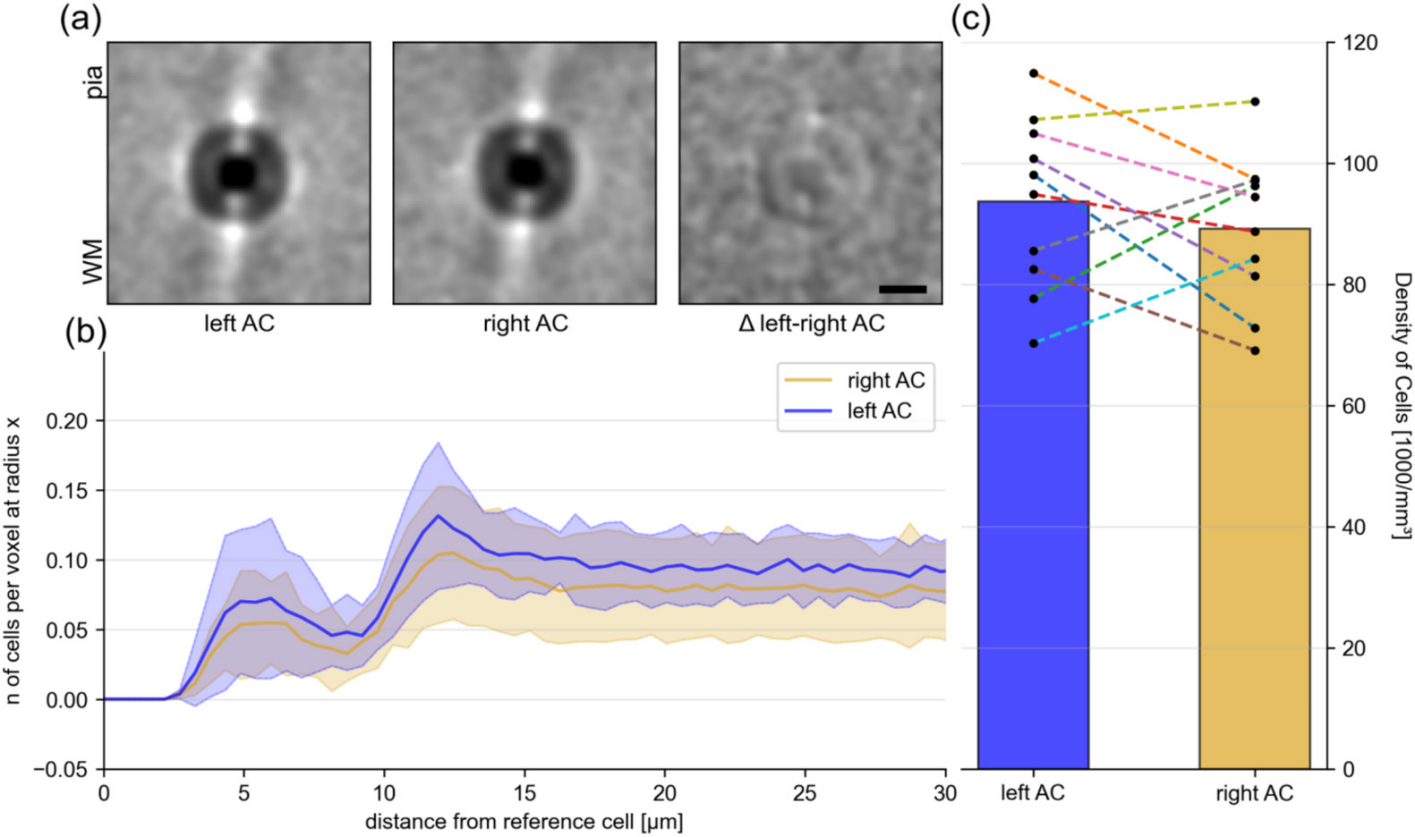
Neuronal cell distributions are largely symmetric in the left and right auditory cortex. (a) Average spatial neighbourhood of every cell in the left and right AC, and the difference between them. The position of the reference cell is in the middle of each panel. All panels are oriented with the cortical surface (pia = pia mater) towards the top and towards white matter (WM) towards the bottom. Scale bar equals 10 μm. (b) The quantity of cells at specific distances to each cell in the left and right AC. The data shown in (b) is normalised towards the volume of voxels at a given radius, meaning the data is probabilistic and not absolute. Shaded areas equal standard error of the mean across all measurements. (c) The mean neuronal cell body density in the left and right AC across all individuals. Bar plots show average of left and right AC, respectively. Points are paired data of each brain sample, with individually corresponding colours as in Figure 2.

In both left and right AC, we annotated neuronal cell bodies in the HuC/HuD channel (Figure 1a) using a computer vision method we term Gabor Spherical Shell filtering (based on Rhodes and Bai 2011). In brief, we convolve a three‐dimensional hollow lens‐shaped kernel (Figure S4) on the raw data and annotate local maxima in the resulting image. From these local maxima (i.e., cell centres), we calculate the average neighbourhood over all annotated cells. We find a clear columnar structure of neuronal cell bodies in both ACs, which is not lateralized (Figure 3a) in males or females (Figure S7). There are two distinct peaks where cells cluster above and below (along the axis from cortical surface to white matter), one at ~6 μm and one at ~12 μm (Figure 3b). We did not find a difference in the density of neurons in the left and right AC (Figure 3c).

## 4 Discussion

Communication sound processing in the mouse AC is lateralized (Ocklenburg, Ströckens, and Güntürkün 2013; Levy et al. 2019). In this study, we find hemispheric asymmetries in intracortical myelin orientation that may explain mechanisms underlying this lateralization. Additionally, we describe different patterns of asymmetry in male and female mice. In contrast to myelin, neuronal cell body distributions are not asymmetric.

Cortical columns are the basic functional unit of cortical processing (Hubel and Wiesel 1963; Markram et al. 2015; Mountcastle 1957; Szentágothai 1975). By analysing the spatial organisation of all neurons (irrespective of subtype), we show that neuronal cell bodies form microcolumns with the same spatial organisation in the left and right AC of mice. This integrates well with the existing literature, as neural progenitors migrate and bud along the same axis in cortical development (Rakic 1972; Tyler et al. 2015), and the resulting neurons are clonally related and have similar functional properties (Li et al. 2012). Because this developmental process is highly conserved in mammals, the symmetric columnar organisation matches our expectations. We also find that both left and right AC show a similar portion of neurons that cluster closer together than typical pyramidal cell distances. We assume that these cells are mostly interneurons, as they tend to cluster closer together than other neurons (Brown et al. 2011; Ebina et al. 2014; Zhang et al. 2017) and, in contrast to excitatory neurons, do not proliferate from radially migrating neural progenitor cells (Tan and Shi 2013).

The majority of neuronal output from AC connects to different neurons within the same column (Wallace, Kitzes, and Jones 1991), forming local neuronal ensembles (See et al. 2018). This is also reflected in myelinated axons in the left and right AC, where we show the majority of connections to run orthogonal to the cortical surface. A subset of neurons connects to neighbouring columns, both within isofrequency bands and along the tonotopic axis (Read, Winer, and Schreiner 2002). These intercolumnar connections from parvalbumin+ and L1 inhibitory interneurons shape sparse representations of stimuli in AC (Liang et al. 2019) and receive input predominantly from neighbouring columns (Tasaka et al. 2023). Since a large part of intracortical myelination stems from myelinated fast‐spiking interneurons (Call and Bergles 2021; Micheva et al. 2016), we can assume that a majority of myelinated axons examined in this study are interneuronal ramifications. The input to inhibitory interneurons in L2/3 largely stems from neighbouring columns (Tasaka et al. 2023), and the same is likely the case for their output: We demonstrate a wider HPD interval of myelin orientations (and therefore more intercolumnar connections) within L2/3 than L4 and L5 in both left and right AC. This suggests that L2/3 is involved in intercolumnar integration of stimuli in AC. Intercolumnar connections along the tonotopic axis might be part of frequency sweep detectors (Levy et al. 2019). This integrates well with functional studies showing wider response properties in L2/3 than L4 (Guo et al. 2012; Winkowski and Kanold 2013), and responses in L2/3 being heterogeneous, even to pure tones (Rothschild, Nelken, and Mizrahi 2010). Inhibitory activity also shapes on and off responses to tones. For example, Liu et al. (2019) show interneuron‐dependent representations of tone onset and offset in the left AC of mice. The symmetric distribution of neurons in the left and right AC shown here suggests no hemispheric differences in the processing of these simple sound features in mice.

However, intercolumnar detection circuits for other sound features may exist in AC and may show hemispheric differences. We checked for structural evidence for two of such circuits. First, we searched for structural analogues to functional circuits described by Levy et al. (2019) in our data. They described a detection circuit for downward frequency sweeps, a common feature of mouse vocalisations, in the left AC, and a more general frequency sweep detecting circuit in the right AC of male mice (females were not tested). We checked for structural asymmetries in fibre orientation along the tonotopic axis in the directions described in their functional data but found no clear evidence corroborating this hemispheric difference. Second, Neophytou et al. (2022) described a temporal integration window difference between left and right AC, based on higher recurrent activity within Layer 2/3 of the right AC. The increased intercolumnar connections in L2/3, L4 and L5 of the right compared to the left AC found in our study may be part of such a circuit.

The described recurrent circuit serves as a potential explanation of a central aspect of human AC lateralization. Asymmetric sampling frequencies of left and right AC were previously described in humans (e.g., Albouy et al. 2020; Poeppel 2003) and may underlie observed hemispheric differences in spectral and temporal processing (e.g., Schönwiesner, Rübsamen, and Yves Von Cramon 2005; Zatorre 2022; Zatorre and Belin 2001). Early auditory areas of mice and humans are more similar in their function than higher order areas for language and acoustic communication (Ruthig and Schönwiesner 2022). Similar circuits may thus exist in humans: Using fluorescent tracers, Galuske and colleagues (Galuske et al. 2000) describe connections to neighbouring cortical patches in human temporal gyrus. Similar to our findings, they find a majority of long‐range (> 3 mm) intercolumnar connections in supragranular layers of the cortex. Although in this study, the spacing of connections was wider in the left AC—which we did not find in mice. Another study showed higher frequency selectivity of voxelwise functional connectivity in the right then left primary AC (Cha, Zatorre, and Schönwiesner 2016), which is suggestive of wider intercolumnar connectivity, in line with the present findings. Therefore, we hypothesise that both sets of findings from mice apply to humans and vice versa. This would mean that both species feature a combination of bottom‐up feature selectivity within AC, which is modulated by top‐down input from higher order areas. These lateralized representations of spectrotemporal features arise from local connectivity within supragranular cortical layers of AC.

The above‐mentioned studies on frequency sweep detectors (Levy et al. 2019) and integration windows in mouse AC (Neophytou et al. 2022) were conducted in male mice. Therefore, strictly speaking, the findings also apply to male mice only. We found that, even on the basic structural level of hemispheric differences in myelinated fibre orientations, AC is very heterogeneous between males and females. The difference between sexes we describe here is larger than intrasexual asymmetry of AC myelin orientation. These differences in the mean direction of the projections may be interpreted as sex‐specific structural specialisations, resulting in different representations of the same stimulus depending on intercolumnar projections, similar to Levy et al. (2019). These sex differences may be rather related to the reception of vocalisations.

Therefore, while vocalisations of male and female mice are relatively similar (Hammerschmidt et al. 2012), we expect fundamental differences in the representation of the same stimulus depending on the sex of the recipient. While functional lateralization of mouse AC is well established, an even larger sex‐dependant asymmetry of a relatively basic anatomical feature such as intracortical myelination is especially noteworthy. It is thus important to include animals of both sexes in future studies of hemispheric asymmetry.

Due to the indirect measurement of connectivity through measuring myelin sheaths (instead of specifically staining, e.g., axons), the statements made in this study cannot be transferred to unmyelinated connections, which make up the majority of intracortical signalling (Call and Bergles 2021). Additionally, the resolution of the given method does not allow for segmentation of single fibres and therefore also does not allow single fibre statistics. Therefore, a more complete description of local circuitry within AC requires further investigation. We chose to analyse the orientation of cortical fibres at the pixel level of the microscopy images. This is the more naive and unbiased approach, as we do not need to segment fibres and have a heavy pre‐processing. However, an alternative would be to segment structures and estimate orientations at the level of individual segments rather than individual pixels. Using segments such as midlines avoids the problem that thick fibres are given more weight in the statistics, but raises the question of how to analyse orientations in the context of cortical layers and the tonotopic axis. In addition, segmentation requires the identification and extraction of midlines, and it is necessary to weight segments (e.g., by length) to ensure fair contributions to the analysis.

## 5 Conclusion

In this study, we show that intracortical myelin orientation in AC is hemispherically asymmetric, depending on cortical layers and sex. We highlight L2/3 being the central hub for intercolumnar signals and the right AC showing more intercolumnar connections in general. However, very basic developmentally defined structures such as cortical columns are not subject to specialisation for different functions in left and right AC. These results suggest a notion of AC where basic structure is retained in both left and right AC, but structural specialisation of the orientation of myelinated fibres enables specific lateralized tasks, such as vocal communication processing, and specialisation on spectral or temporal complexity of stimuli. Furthermore, the reported differences in myelin orientation between male and female mice highlight the importance of including both sexes in studies of auditory signals and vocal communication in particular.

## Author Contributions

**Philip Ruthig:** conceptualization (equal), data curation (equal), formal analysis (equal), investigation (equal), methodology (equal), resources (equal), software (equal), validation (equal), visualization (equal), writing – original draft (equal). **Gesine Fiona Müller:** data curation (equal), formal analysis (equal), investigation (equal), methodology (equal), software (equal), validation (equal), visualization (equal), writing – original draft (equal). **Marion Fink:** data curation (equal). **Nico Scherf:** supervision (equal). **Markus Morawski:** data curation (supporting), formal analysis (supporting). **Marc Schönwiesner:** conceptualization (equal), funding acquisition (equal), project administration (equal), supervision (equal).

## Ethics Statement

All experiments were approved by the provincial regulating office (Landesdirektion Sachsen, Versuchsvorhaben T13/19).

## Consent

The authors have nothing to report.

## Conflicts of Interest

The authors declare no conflicts of interest.

### Peer Review

The peer review history for this article is available at https://www.webofscience.com/api/gateway/wos/peer‐review/10.1111/ejn.16675.

## Supporting information


**Table S1:** Overview of antibodies and staining compounds.

**Table S2:** Lab reagents used in this study, excluding antibodies and staining compounds.

**Figure S1:** Control stainings of autofluorescence (488 nm), HuC/HuD (555 nm), TO‐PRO‐3 (640 nm) and MBP (785 nm) channels. The first row shows a control staining with only first antibodies. The second row shows a control staining with only secondary antibodies (or staining reagent). Images were acquired with a 12× objective (same settings as for the detailed acquisition).
**Figure S2:** Definition of cortical layers. Cortical layers were defined according to expert annotation.
**Figure S3:** Gabor spherical shell projections. This filter kernel was generated using the above‐mentioned equation with following parameters: $r_{0}=22$, σ=12, φ=3.7, $f_{0}=0.1$. Additionally, the Z‐axis of the kernel was compressed by factor 2. A: XY plane. B: YZ plane. C: XZ plane. D: A, B and C combined into a three‐dimensional cross‐section plot.
**Figure S4:** Quantification of cell detection accuracy. A section of the Dataset was cropped and cells were labelled manually in order to establish a ground truth, which was then compared to cells automatically annotated in the original data (A) and the same data with substantial Gaussian noise added (B) in order to estimate the robustness of cell detection. Resulting accuracy is shown in the bar plot (C). False positive % equals to the percentage of cells which were marked as cells by the algorithm, but not in the manual annotation. False negative % equals to the percentage of cells marked in the manual annotation, but not in the algorithm. Accuracy % equals to the percentage of correctly marked cells.
**Figure S5:** Local fibre orientation in anterior, middle and posterior section of AC. Both columns show the distribution of all dominant directions for all female (left) and male (right) samples and included cortical layers (L2/3, L4, L5) for the left and right AC, respectively.
**Figure S6:** Myelin directionality analysis pipeline. The myelin channel (a) was processed using a median and Sato filter and normalised. Then, the structured tensor was computed for each pixel in a sliding window of size 24 × 24 pixels resulting in dominant orientations for each window (c). The autofluorescence channel was used to define the location of each dominant direction with respect to the cortical surface and thus its belonging to a particular cortical layer (b). (d) The mean intensity projection along the z axis of the data for one sample. Orientations are between 0 and 180° and coloured, respectively.
**Figure S7:** Sex differences in neuronal density. These plots show the data from Figure 3c split by sex.

## Data Availability

All pre‐processed (i.e., re‐saved to 3D tiff files) datasets used in this study are freely available on the BioImage Archive database (Sarkans et al. 2018) under the accession number S‐BIAD1077 (www.ebi.ac.uk/biostudies/bioimages/studies/S‐BIAD1077). The script used to re‐save the data from raw 2D files to 3D tiffs is also included in the data repository. All codes necessary to reproduce the results of this paper are available at https://github.com/PhilipRuthig/AC_hemispheric_asymmetry.
