# Supplementary material for: Hemispheric Asymmetry of Intracortical Myelin Orientation in the Mouse Auditory Cortex
Source: Eur J Neurosci. 2025 Jan 20;61(2):e16675. doi: 10.1111/ejn.16675 (PMC11744913; doi:10.1111/ejn.16675)
Supplement: Supplementary file 1 — Table S1: Overview of antibodies and staining compounds. [file EJN-61-0-s002.docx]

| **Name** | **Host** | **Clonality** | **Supplier** | **Catalogue #** | **Lot #** |
| --- | --- | --- | --- | --- | --- |
| HuC/HuD | Mouse | Monoclonal | Invitrogen | A-21271 | 2228014 |
| Myelin Basic Protein | Rat | Monoclonal | Abcam | Ab7349 | GR3360328-2 |
| Cy3 anti-mouse | Donkey | Polyclonal | Jackson Immuno | 715-165-150 | 140334 |
| DyLight 755 anti-rat | Donkey | Polyclonal | Invitrogen | SA5-10031 | VI3079012 |
| TO-PRO-3 | n.a. | n.a. | Invitrogen | T3605 | 1976612 |

Table ST1. Overview of antibodies & staining compounds.
