# Supplementary material for: Hemispheric Asymmetry of Intracortical Myelin Orientation in the Mouse Auditory Cortex
Source: Eur J Neurosci. 2025 Jan 20;61(2):e16675. doi: 10.1111/ejn.16675 (PMC11744913; doi:10.1111/ejn.16675)
Supplement: Supplementary file 2 — Table S2: Lab reagents used in this study, excluding antibodies and staining compounds. [file EJN-61-0-s003.docx]

| **Lab Chemical** | **Supplier** | **Catalogue #** | **Lot #** |
| --- | --- | --- | --- |
| Roti-PreMix PBS | Roth | 0890.1 | 049279863 |
| NaCl | Roth | 3957.3 | K42188704123 |
| Heparin | Ratiopharm | PZN-3029843 | G40338 |
| Paraformaldehyde | Merck | 30525-89-4 | S5679115528 |
| Glutaraldehyde | Merck | 1.04239.0250 | ZC864839927 |
| MeOH | Fisher Scientific | 67-56-1 | 1811301875 |
| H_2_O_2_ 30% | Fluka Chemika | 95300 | 432662/1 22902 |
| Triton X-100 | Fluka Chemika | 93418 | 1249005 15005454 |
| Gelatine | VWR Chemicals | 24350.262 | 18L134106 |
| Dichloromethane | Sigma-Aldrich | 270997 | STBH8667 |
| Benzyl alcohol | thermo scientific | 148390010 | A0439457 |
| Benzyl benzoate | thermo scientific | 105860010 | A0446559 |
| NaN_3_ | Serva | 30175 | 30175 |

Table ST2: Lab reagents used in this study, excluding antibodies & staining compounds.
