# Supplementary material for: Hemispheric Asymmetry of Intracortical Myelin Orientation in the Mouse Auditory Cortex
Source: Eur J Neurosci. 2025 Jan 20;61(2):e16675. doi: 10.1111/ejn.16675 (PMC11744913; doi:10.1111/ejn.16675)
Supplement: Supplementary file 3 — Figure S1: Control stainings of autofluorescence (488 nm), HuC/HuD (555 nm), TO‐PRO‐3 (640 nm) and MBP (785 nm) channels. The first row shows a control staining with only first antibodies. The second row shows a control staining with only secondary antibodies (or staining reagent). Images were acquired with a 12× objective (same settings as for the detailed acquisition). Figure S2: Definition of cortical layers. Cortical layers were defined according to expert annotation. Figure S3: Gabor spherical shell projections. This filter kernel was generated using the above‐mentioned equation with following parameters: r0=22, σ=12, φ=3.7, f0=0.1. Additionally, the Z‐axis of the kernel was compressed by factor 2. A: XY plane. B: YZ plane. C: XZ plane. D: A, B and C combined into a three‐dimensional cross‐section plot. Figure S4: Quantification of cell detection accuracy. A section of the Dataset was cropped and cells were labelled manually in order to establish a ground truth, which was then compared to cells automatically annotated in the original data (A) and the same data with substantial Gaussian noise added (B) in order to estimate the robustness of cell detection. Resulting accuracy is shown in the bar plot (C). False positive % equals to the percentage of cells which were marked as cells by the algorithm, but not in the manual annotation. False negative % equals to the percentage of cells marked in the manual annotation, but not in the algorithm. Accuracy % equals to the percentage of correctly marked cells. Figure S5: Local fibre orientation in anterior, middle and posterior section of AC. Both columns show the distribution of all dominant directions for all female (left) and male (right) samples and included cortical layers (L2/3, L4, L5) for the left and right AC, respectively. Figure S6: Myelin directionality analysis pipeline. The myelin channel (a) was processed using a median and Sato filter and normalised. Then, the structured tensor was computed for each pixel [file EJN-61-0-s001.docx]

**Supplementary Material**

**
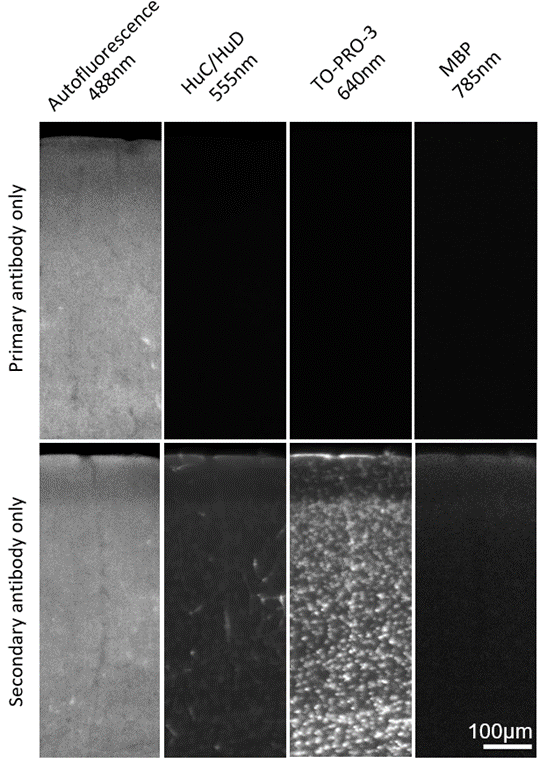
**

**Figure S1: Control stainings of Autofluorescence (488nm), HuC/HuD (555nm), TO-PRO-3 (640nm), and MBP (785nm) channels.** The first row shows a control staining with only first antibodies. The second row shows a control staining with only secondary antibodies (or staining reagent). Images were acquired with a 12x objective (same settings as for the detailed acquisition).


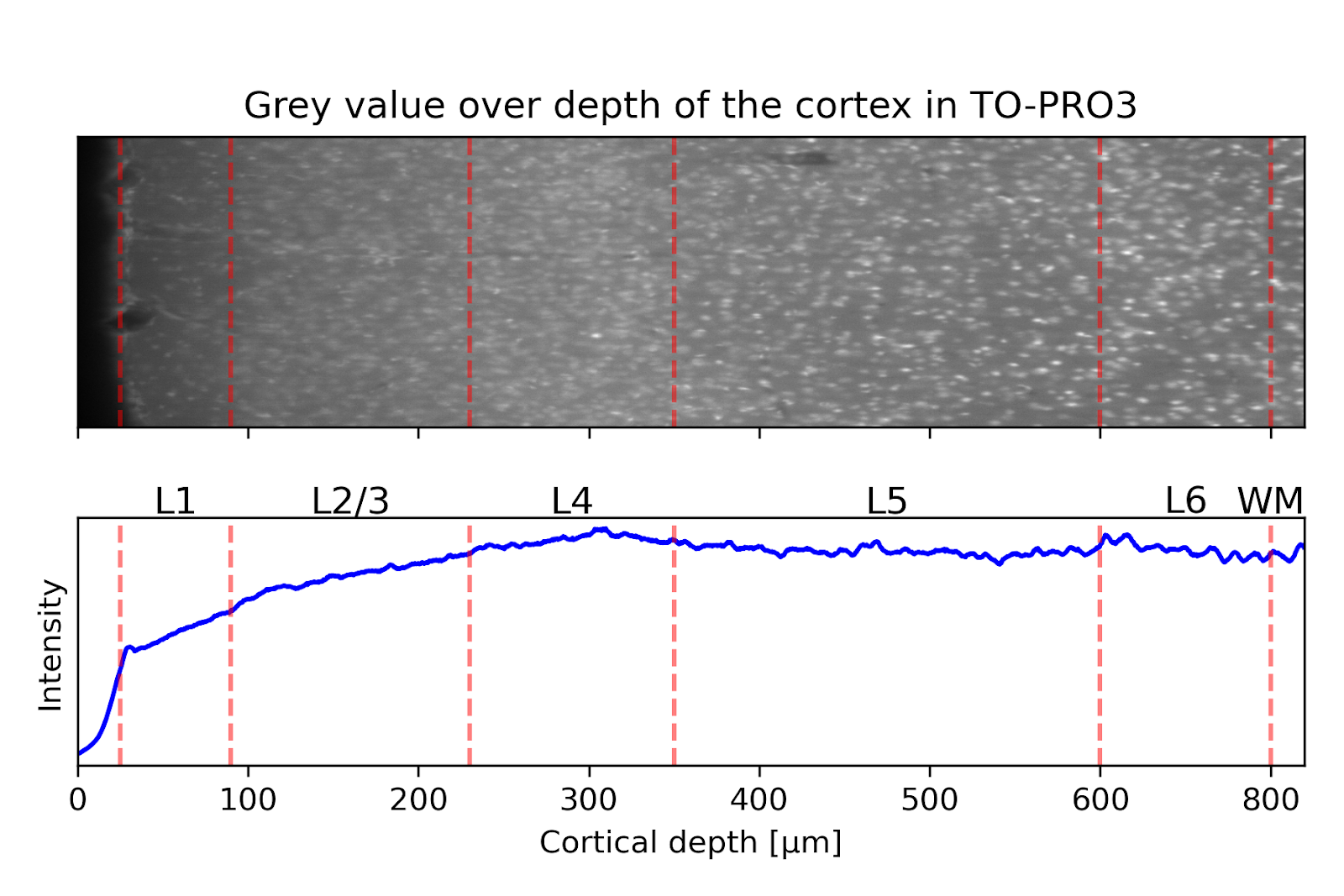


**Figure S2: Definition of cortical layers.** Cortical layers were defined according to expert annotation.

**
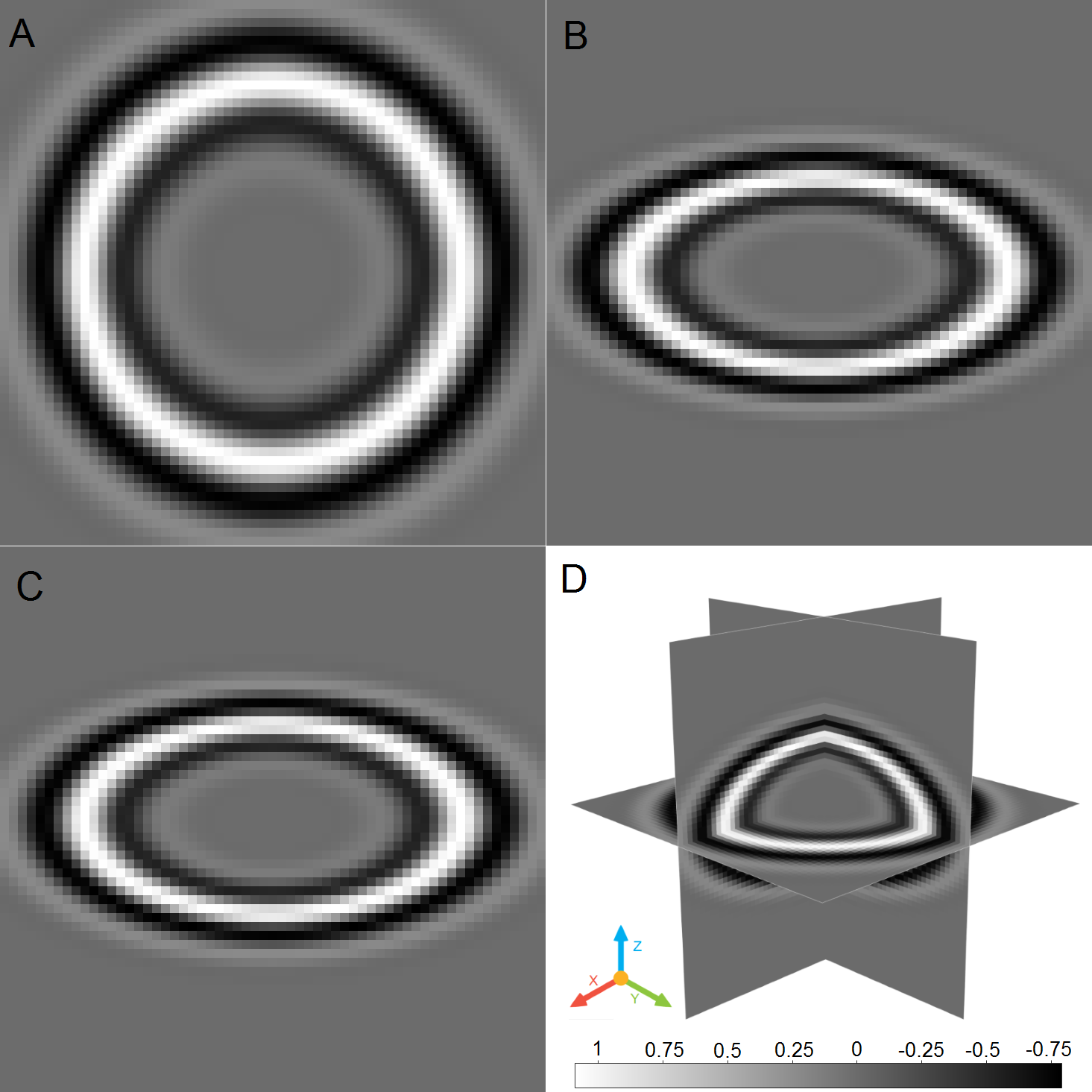
**

**Figure S3: Gabor spherical shell projections.** This filter kernel was generated using the above-mentioned equation with following parameters: $r_{0}=22$,$\sigma=12$, $\varphi=3.7$, $f_{0}=0.1$. Additionally, the Z-axis of the kernel was compressed by factor 2. **A**: XY plane. **B**: YZ plane. **C**: XZ plane. **D**: **A**,**B**,**C** combined into a three-dimensional cross-section plot.

**
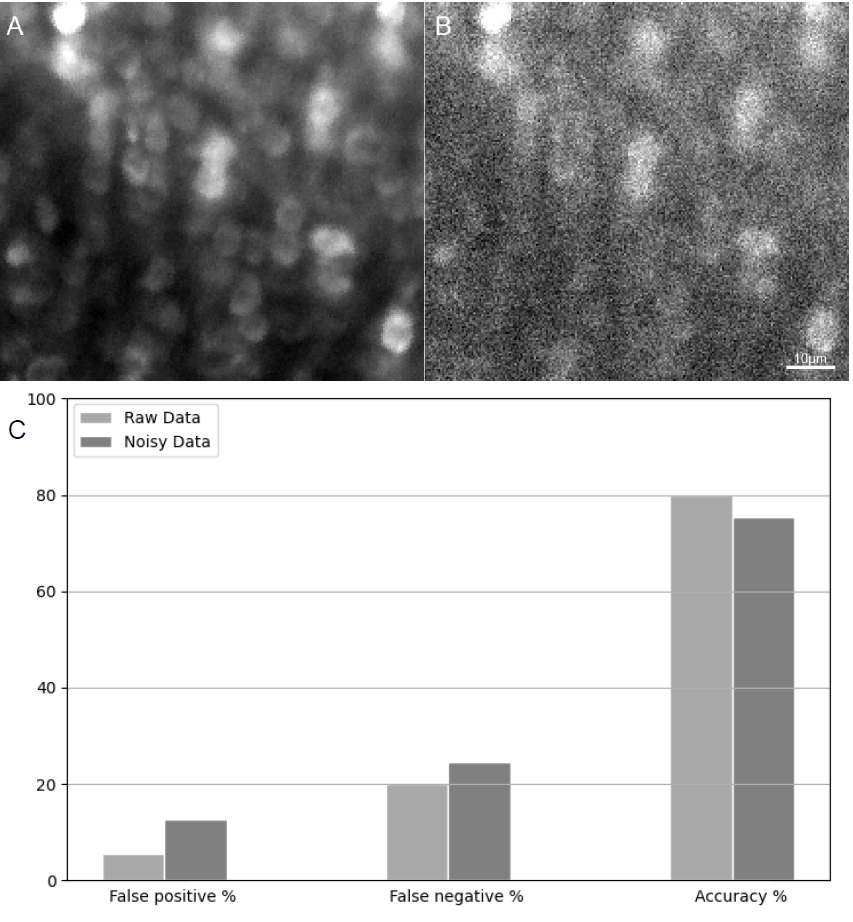
**

**Figure S4: Quantification of cell detection accuracy.** A section of the Dataset was cropped and cells were labelled manually in order to establish a ground truth, which was then compared to cells automatically annotated in the original data (**A**) and the same data with substantial Gaussian noise added (**B**) in order to estimate the robustness of cell detection. Resulting accuracy is shown in the bar plot (**C**). False positive % equals to the percentage of cells which were marked as cells by the algorithm, but not in the manual annotation. False negative % equals to the percentage of cells marked in the manual annotation, but not in the algorithm. Accuracy % equals to the percentage of correctly marked cells.


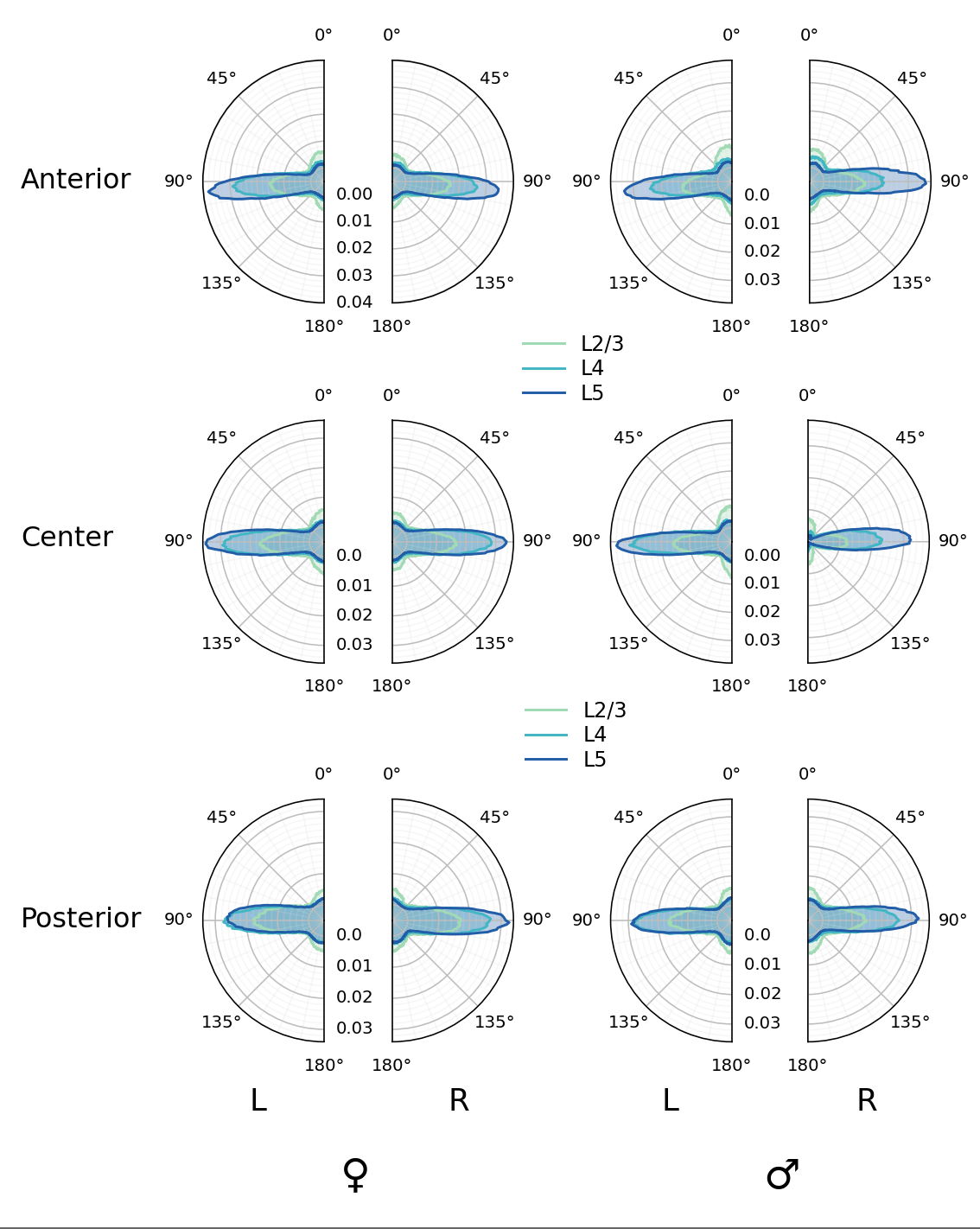


**Figure S5: Local fiber orientation in anterior, middle, and posterior section of AC.** Both columns show the distribution of all dominant directions for all female (left) and male (right) samples and included cortical layers (L2/3, L4, L5) for the left and right AC, respectively.


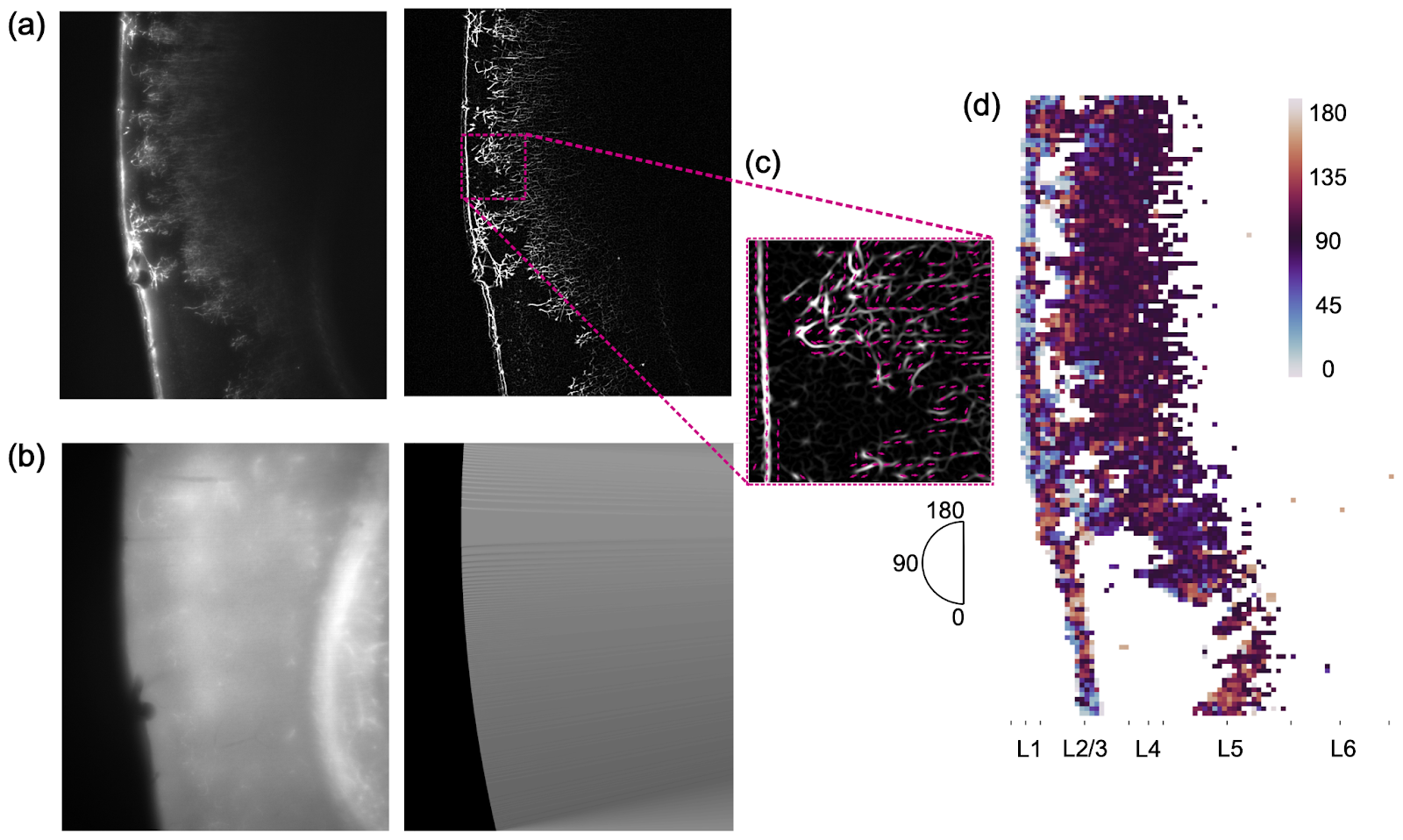


**Figure S6: Myelin directionality analysis pipeline.** The myelin channel **(a)** was processed using a median and Sato filter, and normalized. Then, the structured tensor was computed for each pixel in a sliding window of size 24x24 pixels resulting in dominant orientations for each window **(c)**.The autofluorescence channel was used to define the location of each dominant direction with respect to the cortical surface, and thus its belonging to a particular cortical layer **(b)**. **(d)** shows the mean intensity projection along the z axis of the data for one sample. Orientations are between 0 and 180 degrees and colored respectively.


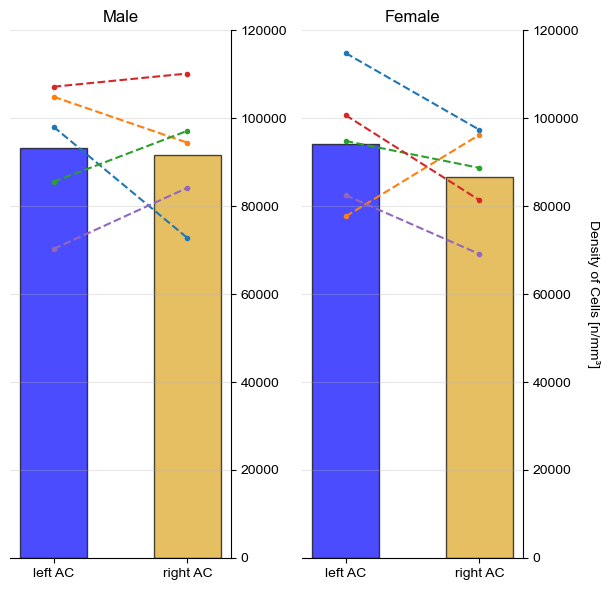


**Figure S7: Sex differences in neuronal density.** These plots show the data from Fig. 3c split by sex.
